# Topographic Analysis of the Isthmus in Mesiobuccal and Mesial Roots of First Molars in a South Korean Population

**DOI:** 10.1038/s41598-020-58364-1

**Published:** 2020-01-27

**Authors:** Sumi Kang, Hui-Wen Yu, Yooseok Shin, Bekir Karabucak, Sunil Kim, Euiseong Kim

**Affiliations:** 1 Microscope Center, Department of Conservative Dentistry and Oral Science Research Center, College of Dentistry, Yonsei University, Seoul, South Korea; 2 Department of Conservative Dentistry, National Health Insurance Ilsan Hospital, Goyang, South Korea; 3 Department of Endodontics, School of Dental Medicine, University of Pennsylvania, Philadelphia, Pennsylvania USA

**Keywords:** Microscopy, Biotechnology

## Abstract

The purpose of this study was to evaluate the incidence and microscopic anatomy of the isthmus to provide more precise anatomical information about the mesiobuccal (MB) roots of the maxillary first molars and the mesial (M) roots of the mandibular first molars. Twenty-eight maxillary and 31 mandibular first molars were embedded, sectioned, stained, and observed at 30× magnification to evaluate the incidence and microscopic anatomy of the isthmus. The incidence of an isthmus 3 mm from the apex was 89.3% and 100% in the MB roots of the maxillary first molars and in the M roots of the mandibular first molars, respectively. The mean dentin thickness between the isthmus and the distal root surface was <1 mm at a distance of 3 mm from the apex in both types of roots. In this study, whenever two main canals were located in the MB roots of the maxillary first molars and in the M roots of the mandibular first molars, the likelihood of the presence of an isthmus increased. Therefore, clinicians should be aware of the thinnest dimensions in the distal surface of the MB roots of the maxillary first molars and the M roots of the mandibular first molars during nonsurgical and surgical root canal treatment.

## Introduction

Endodontic microsurgery is a treatment option when nonsurgical root canal treatment fails to resolve periapical pathosis because of anatomical complexities. Although the success rate of endodontic microsurgery for molars is high, previous studies investigating success rates have reported lower success rates for molars than for anterior teeth^1,2^. The low success rate may be attributed to the limited accessibility of the site and the complexities of root canal anatomy, particularly the presence of an isthmus, which can act as a reservoir for necrotic tissue and bacterial growth^3^. The recognition and management of isthmus areas are important factors that may improve the success rate of endodontic microsurgery in molars^4^.

One of the main anatomical complexities of the molars is the presence of an isthmus, which is defined as a narrow, ribbon-shaped communication between two root canals that contains pulp tissue^5^. Several studies have reported the incidence of an isthmus in the mesiobuccal (MB) roots of the maxillary first molars to range from 76% to 100%, and the incidence has been reported to be approximately 83% in the mesial (M) roots of the mandibular first molars^5–8^. Additionally, a high incidence of an isthmus in the roots of the first molars, particularly located 3 mm to 5 mm from the apex, has also been reported^9^.

In a recent retrospective clinical study, we reported that the success rate of endodontic microsurgery for isthmus-absent teeth was higher than that for isthmus-present teeth in molars^10^. In that study, the most frequent cause of failure identified during extraction or root amputation was vertical root fracture. Sathorn *et al*.^11^ reported that fracture susceptibility increased as the thickness of the remaining root dentin after canal preparation decreased. Additional reductions in root dentin are required for isthmus-present teeth during surgical root-end preparation; thus, further root weakening is unavoidable in isthmus-present teeth compared with isthmus-absent teeth.

Finite element analyses and histologic studies have reported that a root with an isthmus is more likely to be susceptible to fracture than a root without an isthmus^11–13^. A finite element analysis study also found that stress increased when the amount of residual dentin was extremely thin^14^. Thus, we contemplated whether topographic analysis would be helpful for determining the correlation between isthmus preparation and root weakening.

Although the incidence of an isthmus in the first molars has been widely studied^5–9^, studies using topographic measurements of the isthmus of MB roots of the maxillary first molars and of M roots of the mandibular first molars are scarce. Therefore, the purpose of this study was to evaluate the incidence of an isthmus and to assess the microscopic anatomy―minimum dentin thickness, in particular―of root canal systems to provide more precise anatomical information about MB roots of the maxillary first molars and M roots of the mandibular first molars.

## Materials and Methods

### Specimen preparation

Twenty-eight maxillary and 31 mandibular first molars were obtained from December 2016 to April 2017, regardless of the sex or age of the patients, according to protocols approved by the Institutional Review Board (IRB) of National Health Insurance Service, Ilsan Hospital (Gyeonggi-do, South Korea, IRB #2017-04-049). All experiments were performed in accordance with the guidelines and regulations of the IRB of National Health Insurance Service, and informed consent for human-derived materials was obtained from all participants. All teeth were extracted for periodontal or prosthodontic purposes. Teeth with previous endodontic treatments, immature roots, root fractures, or root resorption were excluded. The teeth were sectioned from their crowns in the furcation region using an ultra-thin separating disk (Superdiaflex, HORICO, Berlin, Germany). The teeth were cleaned using an ultrasonic instrument and placed in a 5.25% sodium hypochlorite solution for 1 h to remove remnants of the periodontal ligament. The teeth were dried with air, and the pulp chamber was sealed with utility wax (Atria Corp., Seoul, Republic of Korea) to prevent embedding resins from entering the root canal system.

Each root was separately embedded in clear resin (Ortho-Jet^TM^, LANG, Wheeling, WV). The embedded roots were cut perpendicular to the long axis of the root 1, 2, 3, 4, and 5 mm from the anatomical apex using a low-speed diamond saw (Norton diamond & cBN wheels, Worcester, MA) mounted on a diamond cutter (RB 205 Metsaw-LS, R&B Corp., Daegu, Republic of Korea). The cuts (1 mm thick) were made 1 mm from the apex, and 0.5 mm thick cuts were made 2, 3, 4 and 5 mm from the apex because the disk was 0.5 mm thick. Only the coronal side of each slide was evaluated.

Each section was stored in 5.25% sodium hypochlorite for 1 h to remove organic material remaining inside the root canals. The resected surfaces were stained with 2% methylene blue dye (Duksan, Seoul, Republic of Korea), and photographs were captured using a digital camera (3.1M C-Mount CMOS Camera, KOPTIC Corp., Seoul, Republic of Korea) attached to a stereomicroscope (Nikon SMZ745T, Nikon Corp., Tokyo, Japan) at 30× magnification. ImageJ analysis software (NIH, Bethesda, MD) was used to measure the minimum dentin thickness.

### Classification and Incidence of the Isthmus

**Figure 1:**
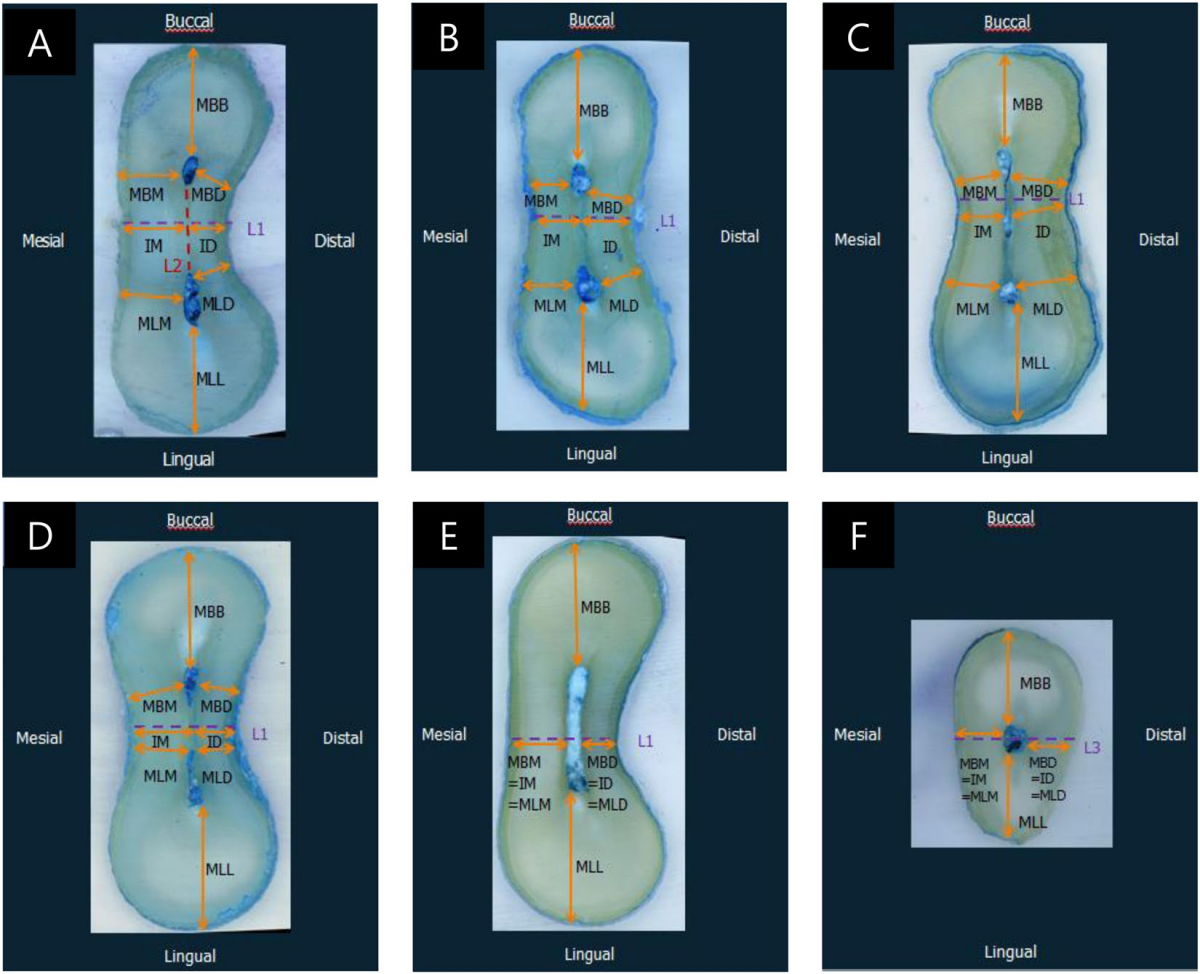
Measurement parameters in each type of isthmus according to the classification reported by Hsu & Kim (5). (**A**) Type I, (**B**) type II, (**C**) type III, (**D**) type IV, (**E**) type V, and (**F**) one canal. L1: The shortest line between the mesial and distal root surfaces. L2: A line between the external lingual limit of the mesiobuccal (MB) canal and the external buccal limit of the mesiolingual (ML) canal. L3: The shortest line between the mesial and distal root surfaces passing through the centre of the canal. MBB: The shortest linear distance between the MB canal wall and the buccal root surface perpendicular to L1. MLL: The shortest linear distance between the ML canal wall and the lingual root surface perpendicular to L1. MBM: The shortest distance between the MB canal wall and the mesial root surface. MBD: The shortest distance between the MB canal wall and the distal root surface. MLM: The shortest distance between the ML canal wall and the mesial root surface. MLD: The shortest distance between the ML canal wall and the distal root surface. IM: Distance from the intersection of L1 and L2 to the mesial root surface. ID: Distance from the intersection of L1 and L2 to the distal root surface.

The type of isthmus configuration was classified according to the report by Hsu and Kim^9^ (Fig. 1). A type I isthmus was defined as either two or three canals with no notable communication. A type II isthmus was defined as two canals that possessed a definite connection between the two main canals. A type III isthmus differed from a type II isthmus only in the presence of three canals instead of two. Canals extending to the isthmus area were classified as a type IV isthmus. A type V isthmus was identified as a true connection throughout the section.

The incidence of an isthmus at each level was recorded based on the classification reported by Teixeira^15^. The Hsu and Kim^9^ classification of a type I isthmus was classified as no isthmus; types II, III, and IV were classified as a partial isthmus; and type V was classified as a complete isthmus.

### Measurement of minimum dentin thickness and statistical methods

The minimum dentin thickness of each specimen was recorded. The measurement parameters according to each type of isthmus are described in Fig. 1. The abbreviations for the measurement parameters are delineated as follows: L1: the shortest line between the mesial and distal root surfaces; L2: a line between the external lingual limit of the mesiobuccal (MB) canal and the external buccal limit of the mesiolingual (ML) canal; L3: the shortest line between the mesial and distal root surfaces passing through the centre of the canal; MBB: the shortest linear distance between the MB canal wall and the buccal root surface perpendicular to L1; MLL: the shortest linear distance between the ML canal wall and the lingual root surface perpendicular to L1; MBM: the shortest distance between the MB canal wall and the mesial root surface; MBD: the shortest distance between the MB canal wall and the distal root surface; MLM: the shortest distance between the ML canal wall and the mesial root surface; MLD: the shortest distance between the ML canal wall and the distal root surface; IM: the distance from the intersection of L1 and L2 to the mesial root surface; and ID: the distance from the intersection of L1 and L2 to the distal root surface.

One-way analysis of variance and multiple comparisons using the Bonferroni post hoc test were conducted to evaluate the minimum dentin thicknesses. All data were analysed using SPSS version 23.0 (IBM Corporation, Somers, NY), and the significance level was established at 0.05.

## Results

### Classification and incidence of an isthmus

A total of 140 and 155 specimens were obtained from the maxillary and mandibular first molars, respectively. The results are summarized in Table 1. In the MB roots of the maxillary first molars, a type II isthmus was the most prevalent at distances of 2 mm, 3 mm and 4 mm from the apex, and a type V isthmus was the most prevalent at a distance of 5 mm from the apex. The incidence of an isthmus was most prominent 3 mm from the apex (89.3%). In the M roots of the mandibular first molars, a type V isthmus was the most prevalent 2 mm, 3 mm, 4 mm, and 5 mm from the apex. The incidence of an isthmus was 100% 3 mm from the apex.

**Table 1:** Overview of the types of isthmus based on the Hus & Kim^5^ classification and the incidence of isthmus based on the Teixeira^12^ NI; no isthmus (no canal + one canal + type I isthmus), PI; partial isthmus (type II + III + IV isthmus), CI; complete isthmus (type V isthmus).

	Distance from apex	n	No canal	One canal	Type I	Type II	Type III	Type IV	Type V	NI	PI	CI	PI + CI
										n (%)	n (%)	n (%)	n (%)
MB root of maxillary first molar	1 mm	28	4	11	10	2	1	—	—	25 (89.3%)	3 (10.7%)	—	3 (10.7%)
	2 mm	28	—	4	4	9	6	2	3	8 (28.6%)	17 (60.7%)	3 (10.7%)	20 (71.4%)
	3 mm	28	—	3	—	10	5	6	4	3 (10.7%)	21 (75.0%)	4 (14.3%)	25 (89.3%)
	4 mm	28	—	4	4	7	4	4	5	8 (28.6%)	15 (53.5%)	5 (17.9%)	20 (71.4%)
	5 mm	28	—	1	3	5	3	5	11	4 (14.3%)	13 (46.4%)	11 (39.3%)	24 (85.7%)
M root of mandibular first molar	1 mm	31	7	9	10	2	3	—	—	26 (83.9%)	5 (16.1%)	—	5 (16.1%)
	2 mm	31	—	7	2	4	3	5	10	9 (29.0%)	12 (38.7%)	10 (32.3%)	22 (71.0%)
	3 mm	31	—	—	—	7	2	9	13	—	18 (58.1%)	13 (41.9%)	31 (100%)
	4 mm	31	—	—	1	11	2	5	12	1 (3.2%)	18 (58.1%)	12 (38.7%)	30 (96.8%)
	5 mm	31	—	—	2	6	8	6	9	2 (6.5%)	20 (64.5%)	9 (29.0%)	29 (93.5%)

### Measurement of minimum dentin thickness

Four specimens from the MB roots of the maxillary first molars and seven specimens from the M roots of the mandibular first molars had no canal present within 1 mm from the apex. At the completion of the assessment, 136 specimens from the MB roots of the maxillary first molars and 148 specimens from the M roots of the mandibular first molars were evaluated. The mean minimum dentin thickness in both types of roots for each measurement parameter is presented in Table 2.

**Table 2:** Mean minimum dentin thickness (mm) in the MB roots of the maxillary first molars and M roots of the mandibular first molars. For each parameter, different letters indicate a significant difference (P < 0.05).

	Distance from apex	n	MBB	MLL	MBM	MBD	MLM	MLD	IM	ID
			Mean ± SD	Mean ± SD	Mean ± SD	Mean ± SD	Mean ± SD	Mean ± SD	Mean ± SD	Mean ± SD
MB root of maxillary first molar	1 mm	24	1.00 ± 0.55^a^	0.96 ± 0.53^a^	0.76 ± 0.25^a^	0.69 ± 0.61^a^	0.74 ± 0.28^a^	0.63 ± 0.61^a^	0.78 ± 0.23^a^	0.69 ± 0.58^a^
	2 mm	28	1.37 ± 0.26^b^	0.95 ± 0.52^a^	1.01 ± 0.28^b^	0.91 ± 0.23^a,b^	0.84 ± 0.36^a,b^	0.65 ± 0.24^a^	0.89 ± 0.30^a,b^	0.80 ± 0.23^a,b^
	3 mm	28	1.69 ± 0.28^c^	1.53 ± 0.57^b^	1.13 ± 0.25^b,c^	1.01 ± 0.27^b^	0.96 ± 0.22^b,c^	0.78 ± 0.20^a,b^	1.03 ± 0.23^b,c^	0.87 ± 0.21^a,b^
	4 mm	28	1.90 ± 0.32^c,d^	1.95 ± 0.55^b,c^	1.21 ± 0.22^b,c^	1.05 ± 0.25^b^	1.08 ± 0.21^c^	0.85 ± 0.20^a,b^	1.10 ± 0.25^b,c^	0.95 ± 0.24^a,b^
	5 mm	28	2.03 ± 0.29^d^	2.17 ± 0.47^c^	1.32 ± 0.20^c^	1.13 ± 0.20^b^	1.15 ± 0.17^c^	0.93 ± 0.18^b^	1.22 ± 0.18^c^	1.03 ± 0.22^b^
M root of mandibular first molar	1 mm	24	1.06 ± 0.66^a^	0.85 ± 0.35^a^	0.78 ± 0.52^a^	0.48 ± 0.32^a^	0.77 ± 0.68^a^	0.47 ± 0.33^a^	0.72 ± 0.62^a^	0.51 ± 0.29^a^
	2 mm	31	1.45 ± 0.38^b^	1.54 ± 0.50^b^	0.89 ± 0.25^a,b^	0.80 ± 0.30^b^	0.94 ± 0.29^a,b^	0.77 ± 0.24^b^	0.89 ± 0.24^a,b^	0.76 ± 0.22^b^
	3 mm	31	1.73 ± 0.32^b,c^	1.80 ± 0.44^b,c^	1.04 ± 0.20^b,c^	0.92 ± 0.22^b^	1.06 ± 0.29^b^	0.86 ± 0.25^b^	0.96 ± 0.25^a,b^	0.88 ± 0.23^b^
	4 mm	31	1.87 ± 0.33^c^	1.98 ± 0.40^c^	1.12 ± 0.17^c^	1.00 ± 0.24^b^	1.15 ± 0.22^b^	0.94 ± 0.26^b^	1.07 ± 0.22^b^	0.86 ± 0.27^b^
	5 mm	31	1.96 ± 0.31^c^	2.02 ± 0.39^c^	1.17 ± 0.17^c^	0.97 ± 0.23^b^	1.22 ± 0.18^b^	0.94 ± 0.25^b^	1.10 ± 0.19^b^	0.91 ± 0.25^b^

**Figure 2:**
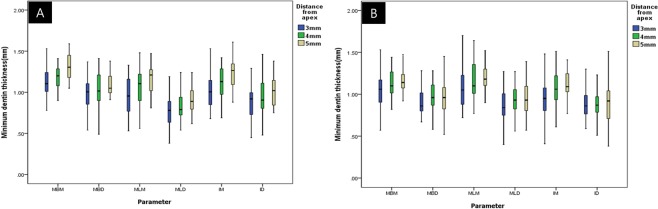
Mean dentin thickness 3 mm, 4 mm, and 5 mm from the apex. (**A**) Mesiobuccal root and (**B**) mesial root. In both roots, no statistically significant differences were found in all parameters defined (P > 0.05).

In the MB roots of the maxillary first molars, the mean value of minimum dentin thickness tended to increase as the distance from the apex increased. There were significant differences in MBB (1 mm < 2 mm < 3, 4, and 5 mm), MLL (1 and 2 mm < 3, 4 and 5 mm), and MBM (1 mm < 2, 3, 4 and 5 mm) (P < 0.05). The dentin thickness increased up to a distance of 3 mm from the apex, but there were no significant differences among the other levels. There were no significant differences in MBD, MLM, MLD, IM and ID (P > 0.05) among the samples from different section levels. Figure 2A illustrates the comparison of the mesiodistal dimension (MBM, MBD, MLM, MLD, IM and ID) in the MB roots of the maxillary first molars 3 mm, 4 mm, and 5 mm from the apex. There were no statistically significant differences among the samples from groups 3 mm, 4 mm, and 5 mm from the apex.

In the M roots of the mandibular first molars, the mean minimum dentin thickness tended to increase as the distance from the apex increased. There were significant differences in MBB (1 mm < 2, 3, 4, and 5 mm), MLL (1 < 2, 3, 4, and 5 mm), MBD (1 mm < 2, 3, 4, and 5 mm), MLD (1 mm < 2, 3, 4, and 5 mm), and ID (1 mm < 2, 3, 4, and 5 mm) (P < 0.05) between the 1 mm and 2 mm levels, but there was no significant difference among the other levels. There were no significant differences in MBM, MLM, or IM (P > 0.05) among samples from different section levels. Figure 2B illustrates the comparison of the mesio-distal dimension (MBM, MBD, MLM, MLD, IM and ID) in the M roots of the mandibular first molars 3 mm, 4 mm, and 5 mm from the apex. There were no statistically significant differences among the samples from groups 3 mm, 4 mm, and 5 mm from the apex.

## Discussion

Several studies have investigated the anatomical structure of the maxillary and mandibular first molar(s) using various methods^5,8,9,16–18^. Micro-computed tomography (Micro-CT) has emerged as a modern technique used for the evaluation of root canal anatomy by reconstructing the root canal system. Although micro-CT has the advantage of not destroying the specimen, the size of an isthmus is too small to obtain an accurate image with micro-CT. Sectioning and staining have greater accuracy than other techniques for the detection of an isthmus. Regardless of whether a particular technique destroys the specimen, however, it is important to accurately characterize the isthmus and measure the minimum dentin thickness. Therefore, we sectioned and evaluated the teeth under a stereomicroscope. To the best of our knowledge, there are no published studies that have used topographic techniques to evaluate the incidence of an isthmus on the MB roots of the maxillary first molars and on the M roots of the mandibular first molars.

In the present study, the incidence of an isthmus in the MB roots of the maxillary first molars and the M roots of the mandibular first molars was 71.4% to 89.3% and 71.0% to 100%, respectively, at the investigated resection levels (except the 1 mm section). These results support the findings of previous *in vivo* research that reported the incidence of an isthmus. Von Arx^8^ reported that, in cases requiring root-end surgery, the incidence of an isthmus 3 mm to 4 mm from the apex in the MB roots of the maxillary first molars and the M roots of the mandibular first molars was 76% and 83%, respectively. The high incidence of an isthmus 2 mm to 5 mm from the apex suggests that these spaces may be a concern during nonsurgical root canal treatment. Due to the complex structure of an isthmus, it is difficult to perform direct mechanical preparation and chemical disinfection. To overcome this anatomical complexity, various preparation and irrigation methods have been introduced to disinfect necrotic debris and tissue remnants in the isthmus^19,20^. Sufficient use of appropriate irrigation fluids, such as sodium hypochlorite, is also required^21^.

The high incidence of an isthmus is also a significant concern in surgical root canal treatment. Modern endodontic surgery suggests that the optimal root-end resection level is 3 mm to 4 mm^22,23^. Whenever two main canals are located in the MB roots of the maxillary first molars and in the M roots of the mandibular first molars, the greater the likelihood is of the presence of an isthmus at the resected root-end surface. Thus, clinicians should be aware of the high incidence of an isthmus, and resected surfaces should be carefully observed at a high magnification using a surgical operating microscope after methylene blue staining.

With nonsurgical root canal treatment, the residual dentin thickness following intra-radicular procedures is correlated with the fracture resistance of the root^24^. It has been suggested that 3 mm is the minimum dentin thickness of canal walls that should remain for canal preparation. This may be the case when performing root-end preparation after root resection in endodontic surgery. Because the shape of the root is normally tapered at the apex, the residual dentin thickness on the resected root can be considered more important than that on the coronal part of the teeth. Additionally, in our previous study^10^, we found that the lower success rate in isthmus-present teeth was associated with the isthmus preparation procedure itself, which resulted in weakening of the root. Therefore, we also observed the minimum dentin thickness at various parameters in each section.

The average ID 3 mm from the apex was 0.87 mm and 0.88 mm (i.e., <1 mm) in the MB roots of the maxillary first molars and in the M roots of the mandibular first molars, respectively. The mean MLD of both types of roots was also <1 mm 3 mm from the apex. These results have important implications for retro-preparation after root-end resection. Considering that the diameter of the ultrasonic tips used in root-end preparation is 0.5 mm, isthmus preparation 3 mm from the apex will leave a thin dentin layer of <0.62 mm and 0.63 mm at the ID in MB roots of the maxillary first molars and in M roots of the mandibular first molars, respectively. Furthermore, misalignment during root-end preparation is a common complication during root-end surgery, which may result in further removal of dentin. Many investigators have raised concerns that ultrasonic tips create micro-fractures in thin areas of the root. Abedi *et al*.^13^ recommended that the remaining dentin thickness after retro-preparation should be at least 1 mm because of a significant increase in the incidence of micro-fractures in dentin <1 mm thick. One textbook of surgical endodontics recommends that a 2 mm border of the peripheral root structure must exist around the cavity preparation; if not, the root should be resected further, in 1 mm increments, until there is 2 mm present^25^. However, in this study, there was no region with a minimum dentin thickness of 2 mm in both roots 1 mm to 5 mm from the apex, even without previous enlargement of the canals during nonsurgical endodontic treatment and retro-preparation. Degerness and Bowels^17^ reported results similar to those of our study. Furthermore, there were no statistically significant differences between the mesio-distal dimension (i.e., MBM, MBD, MLM, MLD, IM and ID) in either roots at distances of 3 mm, 4 mm, and 5 mm from the apex. Therefore, resecting the root more coronally to obtain thicker levels of remaining dentin would be avoided.

These results are also meaningful for nonsurgical root canal treatment. MLD and ID are parameters involved in the “danger zone”, which is prone to perforation when performing root canal treatment. Excessive mechanical preparation for the removal of bacteria and adequate irrigation needle depth would be avoided in both roots, preventing iatrogenic perforation and weakening of the root.

A previous study reported that root canal length varied according to race. Kim *et al*.^26^ reported that the MB root canal length of the maxillary first molars in Asians was 1.5 mm shorter than that in Caucasians. The mean difference in canal length of pooled teeth between the Asian and Caucasian teeth was 1.2 mm. It was difficult to find studies comparing the thickness of roots according to race; nevertheless, it is highly probable that there is a difference in dentin thickness according to race, considering the difference in root canal length. Therefore, the results of this study, which involved an Asian population, may be different in a Caucasian cohort.

One strength of the present study was the measurement of minimum dentin thickness and the description of the type of isthmus, which have not been previously reported. Nevertheless, this study had some limitations, including its relatively small sample size and the fact that the age and sex of the donors were not considered. Within these limitations, however, clinicians must be aware of the thinnest dimensions in the distal surface of MB roots of the maxillary first molars and M roots of mandibular first molars during root-end preparation and must conscientiously plan in the mesial direction, where a relatively abundant tooth structure is present and could be helpful in preventing root weakening. Research investigating new ultrasonic tips that minimize weakening of the remaining dentin at the thinnest area or a new method of root-end preparation that minimizes stress distribution may improve the success and long-term prognosis of endodontic surgery in isthmus-present teeth.

## Conclusions

In conclusion, whenever two main canals were located in the MB roots of the maxillary first molars and in the M roots of the mandibular first molars, the likelihood of an isthmus increased. Clinicians should be aware of the thinnest dimensions in the distal surface of MB roots of the maxillary first molars and the M roots of the mandibular first molars during nonsurgical and surgical root canal treatment.
